# The role of computed tomography in the assessment of tumour extent and the risk of residual disease after upfront surgery in advanced ovarian cancer (AOC)

**DOI:** 10.1007/s00404-022-06466-8

**Published:** 2022-03-02

**Authors:** Mihaela Asp, Susanne Malander, Nils-Olof Wallengren, Sonja Pudaric, Johan Bengtsson, Hanna Sartor, Päivi Kannisto

**Affiliations:** 1 Division of Obstetrics and Gynecology, Department of Clinical Science Lund, Skåne University Hospital, Lund University, Lund, Sweden; 2 Division of Oncology, Department of Clinical Science Lund, Skåne University Hospital, Lund University, Lund, Sweden; 3 Division of Medical Imaging and Physiology, Department of Clinical Science Lund, Skåne University Hospital, Lund University, Lund, Sweden; 4 Diagnostic Radiology, Department of Translational Medicine, Lund University, Skåne University Hospital, Lund, Sweden

**Keywords:** Ovarian cancer, Peritoneal cancer index, Cytoreductive surgery, Computed tomography

## Abstract

**Purpose:**

Epithelial ovarian cancer is usually diagnosed in the advanced stages. To choose the best therapeutic approach, an accurate preoperative assessment of the tumour extent is crucial. This study aimed to determine whether the peritoneal cancer index (PCI), the amount of ascites, and the presence of cardiophrenic nodes (CPLNs) visualized by computed tomography (CT) can assess the tumour extent (S-PCI) and residual disease (RD) for advanced ovarian cancer (AOC) patients treated with upfront surgery.

**Methods:**

In total, 118 AOC cases were included between January 2016 and December 2018 at Skåne University Hospital, Lund, Sweden. Linear regression and interclass correlation (ICC) analyses were used to determine the relationship between CT-PCI and S-PCI. The patients were stratified in complete cytoreductive surgery (CCS) with no RD or to non-CCS with RD of any size. The amount of ascites on CT (CT-ascites), CA-125 and the presence of radiological enlarged CPLNs (CT-CPLN) were analysed to evaluate their impact on estimating RD.

**Results:**

CT-PCI correlated well with S-PCI (0.397; 95% CI 0.252–0.541; *p* < 0.001). The risk of RD was also related to CT-PCI (OR 1.069 (1.009–1.131), *p* < 0.023) with a cut-off of 21 for CT-PCI (0.715, *p* = 0.000). The sensitivity, specificity, positive predictive value and negative predictive value were 58.5, 70.3, 52.2 and 75.4%, respectively. CT-ascites above 1000 ml predicted RD (OR 3.510 (1.298–9.491) *p* < 0.013).

**Conclusion:**

CT is a reliable tool to assess the extent of the disease in advanced ovarian cancer. Higher CT-PCI scores and large volumes of ascites estimated on CT predicted RD of any size.

## Background

Epithelial ovarian cancer is the gynaecological malignancy with the highest mortality rate, with a five-year survival rate below 45% [1, 2]. More than 70% of ovarian cancer cases are diagnosed in the advanced stages with carcinosis in the abdominal cavity [3]. Advanced ovarian cancer (AOC) is generally found in the omentum and peritoneal surfaces from the diaphragm to the pouch of Douglas due to ascites formation and circulation in the abdominal cavity [4].

The standard treatment for AOC is primary debulking surgery (PDS) followed by platinum-based postoperative chemotherapy [5]. Since surgery is the only way to improve prognosis for patients with AOC, the abdominal tumour extent must be well characterized to effectively plan the surgery and achieve maximal radicality. One way to characterize the tumour burden is by numeric quantification of the tumour in the peritoneal cavity by the surgeon, an approach first described by Sugarbaker in 1998 for colorectal cancer [6, 7]. A higher peritoneal cancer index (PCI) indicates a worse surgical outcome and risk of residual tumour. This matter suggests considering treatment options for the patient other than primary surgery [8]. Likewise, PCI estimation from a preoperative CT scan (CT-PCI) is not a component of standard guidelines for ovarian cancer diagnostics, but previous studies on CT-PCI in ovarian cancer have shown a significant relationship between CT-PCI and suboptimal surgery, postoperative complications and overall survival (OS) [9, 10].

Investigators have tried to find the optimal benefit of preoperative CT scans to identify radiological predictors of tumour extent [11]. Along with the development of imaging techniques, markers, such as cardiophrenic lymph nodes (CPLNs), have been identified in patients with AOC. To our knowledge, there is no clear evidence that removing the lymph node leads to better OS or PFS in AOC [12]. CPLN metastases are associated with impaired PFS and OS in patients with AOC [13].

CA-125 is a high-molecular-weight glycoprotein expressed in a large number of diseases and by a large proportion of epithelial ovarian cancer cases, with poor specificity and sensitivity [14]. Several studies have tried to predict surgical outcomes using CA-125 in AOC, with inconsistent results [15–18].

This study aims to investigate whether preoperative imaging (CT-PCI, CT-ascites, and CT-CPLN) is predictive of tumour extent (S-PCI) and residual disease of any size.

## Methods

### Patient characteristics

A total of 194 patients with AOC (FIGO stage III-IV) were diagnosed and treated from January 2016 to December 2018 at Skåne University Hospital, a tertiary centre for gynaecologic cancer treatment in Southern Sweden. Patient data were collected retrospectively from the patients’ medical records. Patients receiving neoadjuvant chemotherapy with interval debulking surgery (33 patients, 17%) or with palliative chemotherapy only (15 patients, 7.7%) were excluded. Twenty-eight patients were excluded due to insufficient data. In total, 118 patients who were deemed suitable for upfront extensive surgery were included in the study.

Clinical data on tumour stage (FIGO), histology type, age, performance status (ECOG: Eastern Cooperative Oncology Group) and CA-125 were collected.

The clinical characteristics of all patients included in the study are shown in Table 1. The group with complete cytoreductive surgery (CCS) and patients with residual tumour (non-CCS) are shown separately.

**Table 1:** Patients’ characteristics

Characteristics	All patients	Non-CCS	CCS	
*N*	118	43 (36.4%)	75 (63.6%)	(*p* < 0.136)
Age				
Mean (range)	67 (28–89)	68.42 (28–29)	66.44 (36–84)	
FIGO stage				
III	90 (76.3%)	31 (72.1%)	59 (78.7%)	(*p* < 0.421)
IV	28 (23.7%)	12 (27.9%)	16 (21.3%)	
Histology				
High-grade serous	88 (83.9%)	67 (89.3%)	32(74.4%)	
Others	19 (16.1%)	8 (10.7%)	11 (25.6%)	
SCA-125 U/mL				(*p* < .036)
Mean (range)	877 (15–10,000)	993.53 (16–4000)	811 (15–10,000)	
S-PCI				(*p* < 0.0001)*
Mean (range)	16.46 (2–28)	22.33 (4–38)	13.04 (2–26)	
S-PCI no (%)				
S-PCI ≤ 10	28 (23.7%)	2 (4.8%)	26 (36.1%)	
S-PCI 11–20	52 (44.2%)	15 (35.7%)	37 (51.4%)	
S-PCI ≥ 20	34 (28.8%)	25 (59.5%)	9 (12.5%)	
CT-PCI				(*p* < 0.001)*
Mean (range)	17.12 (0–26)	21(0–36)	14.84 (0–30)	
CT-PCI no (%)				
CT-PCI ≤ 10	30 (25.4%)	8 (19.5%)	22 (29.7%)	
CT-PCI 11–20	39 (33.1%)	9 (22.0%)	30 (40.5%)	
CT-PCI ≥ 20	46 (39%)	24 (58.5%)	22 (29.7%)	
Missing	3 (2.5%)			
ECOG				
≤ 1	99 (83.9%)	31 (72.1%)	59 (78.7%)	(*p* < 0.42)
≥ 2	19 (16.1%)	12 (27.9%)	16 (21.3%)	
CPLN				
< 5 mm	59 (50%)	18 (42%)	34 (45.3%)	(*p* < .182)
≥ 5 mm	59 (50%)	25 (58%)	41 (54.7%)	

*PCI* peritoneal cancer index, *S-PCI*  surgical PCI, *CT-PCI*  computed tomography PCI, *ECOG* eastern cooperative oncology group-performance status, *FIGO*  eastern cooperation oncology group, *CCS* complete cytoreductive surgery, *CPLN*  cardiophrenic lymph nodes

*P < 0.05 was considered significant

### Assessment of tumour extent (S-PCI) and ascites using CT scans (CT-PCI and CT-ascites) and CA-125

To ensure the accuracy of the surgical documentation and S-PCI, both procedures were performed by the same surgeon for a series of 25 patients in the cohort. The author calculated the S-PCI score for the 25 surgical records, which was compared with the original S-PCI score calculated by the surgeon for each case. The mean and standard deviation (SD) of the original S-PCI was 17.07 ± 9.41, and the newly calculated S-PCI was 16.23 ± 9.03. As the data were normally distributed, Student’s t-test was used to confirm a very good correlation with the original measurement. Based on these findings, the S-PCI in the remaining cases was assessed according to the original surgical reports. The PCI score developed by Sugarbaker (1998) was used to quantify both CT-PCI and S-PCI [6]. The 13 abdominal regions were assessed for tumour content and scored from 0 to 3 depending on the tumour size: 0 points indicated no visible tumour, and 1, 2 or 3 points indicated lesions with maximum diameters of 0.5, 5.0, or > 5 cm, respectively, or confluent lesions with a final score between 1 and 39. The results were analysed as continuous data and categorized into three levels (1–10, 11–20, ≥ 21 points).

To evaluate the possible effect of time on the relationship between CT-PCI and surgery (S-PCI), CT-PCI was assessed within 20 days, between 21 and 40 days and beyond 40 days. The 20-day interval was chosen as a reference according to the actual standardized cancer care pathway in Sweden, which requires a maximum of 24 days until the start of treatment [19].

The volume of the ascites at the surgery was categorized into three groups (< 500 ml, 500–1000 and > 1000 ml). Twenty-three of 118 patients underwent paracentesis because of abdominal swelling or tightness. Data from 10 patients were incomplete.

### Assessment of postoperative residual disease

Operability and resectability were evaluated for each patient according to age, medical history, symptoms, medication, social and nutritional status and feasibility of tumour debulking according to preoperative CT of the abdomen and thorax. All therapeutic decisions were made for each patient by a multidisciplinary team (MDT) consisting of imaging specialists, gynaecologic oncologists, medical oncologists and pathologists. Only patients intended for upfront surgery were included in the study. The surgical outcome was indicated by the amount of residual disease after the surgery. The surgical outcome was stratified into CCS with no residual disease at the end of the surgery and non-CCS, summarizing patients with residual disease of any size.

### Image analysis

**Fig.1:**
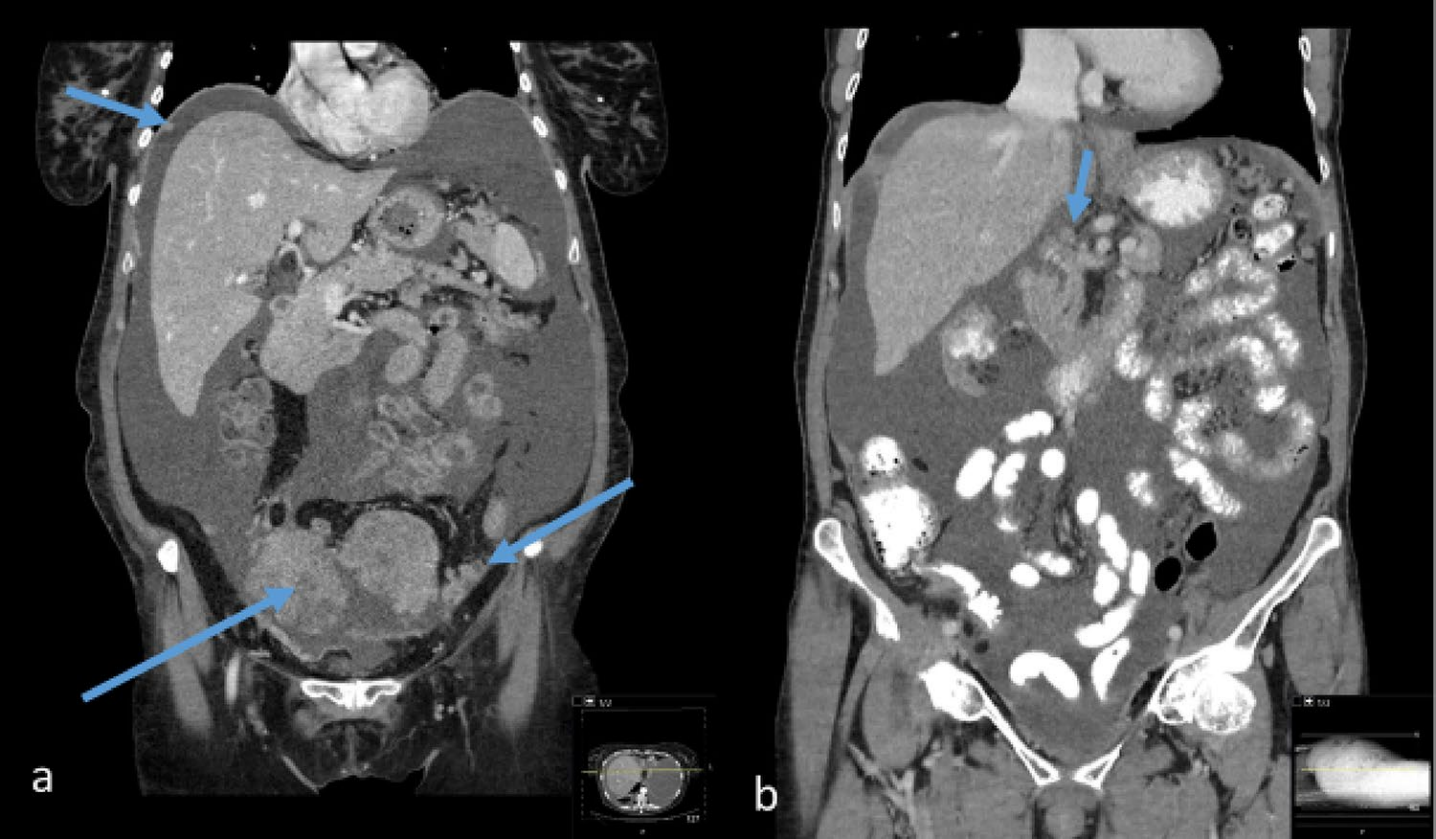
Peritoneal carcinomatosis and ascites in patients with ovarian cancer.** a** CT-image (contrast-enhanced CT of the abdomen and pelvis, coronary projection) showing right diaphragmatic carcinomatosis, left-side pervic carcinomatosis, and pelvic masses. **b** CT-image (intravenous and oral-enhanced CT of the abdomen and pelvis, coronary projection) carcinomatosis in truncus coeliacus area

All eligible patients underwent CT in the supine position with intravenous and oral contrast. Digital CT images were obtained by convention reformatted in the coronal and sagittal planes. CT-PCI was retrospectively scored using the Sugarbaker classification [20] by one of two radiology specialists (HS or JB). The CT-PCI was calculated as the sum of the numerical lesion scores assigned to the 13 abdominopelvic regions and the lesion score to the largest visible implant. Ascites (three groups) was estimated by one of two radiologists (HS or JB) concurrent with the CT-PCI evaluation. CT-ascites was evaluated qualitatively by the interpreting radiologist and assigned to one of three groups (< 500 ml, 500–1000 ml, and > 1000 ml). The CT-ascites evaluation was based on all three image projections. CPLNs were retrospectively assessed by one of two radiology specialists (SP or NOW). CPLN was defined as a pathological enlargement measuring ≥ 5 mm at the short axis in the axial plane and was scored as negative (i.e., normal) or positive (i.e., enlarged) by two radiologists (NOW and SP). The radiologists were blinded to the intraoperative data and surgical outcomes (Fig. 1).

### Statistical analyses

The Stata SE (version 16.0. College Station, Texas: StataCorp) and IBM SPSS Statistics 25 were used for all statistical analyses. For descriptive data, including means, medians and percentages, standard analyses were used. The normality of the data was tested using the one-sample Kolmogorov–Smirnov test. FIGO stage, ECOG score, surgical outcome, and presence of CPLNs were dichotomized and analysed in terms of numbers and percentages. Student’s *t* test and the Mann–Whitney *U* test were used to evaluate the associations of S-PCI and CT-PCI with clinical factors.

Linear regression and interclass correlation (ICC) analyses were used to characterize the agreement between CT-PCI (continuous) and S-PCI (continuous). The data were adjusted for CA-125 level (continuous), ascites (grouped into three categories: < 500, 500–1000, and > 1000 ml) and the number of days between the CT examination and surgery (grouped into three categories: < 20 days, 20–39 days, and ≥ 40 days). An ICC analysis was used to compare S-ascites and CT-ascites (nominal), and weighted kappa and percent agreement were calculated. The kappa value was used to compare the agreement between those two groups (S-ascites and CT-ascites, both divided into three intervals: < 500, 500–1000 and > 1000 ml). A kappa value of 1.00 is reflective of perfect agreement, 0 indicates no agreement, 0.81–1.00 shows very good agreement, and 0.61–0.80 shows good agreement.

Linear and logistic regression analyses were used to determine the relationships between CT-PCI, S-PCI and surgical outcome. Analyses were adjusted for different variables (ascites, CPLNs, CA-125 and days between the CT scan and surgery). Some data were log transformed due to an asymmetric distribution (CA 125).

The receiver operating characteristic curve (ROC) was used to calculate a cut-off for CCS for both CT-PCI and S-PCI.

Linear and logistic regression analyses were used to determine the impact of positive radiological CPLNs on the surgical outcome. We compared the presence or absence of CPLNs, dichotomized and continuous. The results are presented as odds ratios (ORs) with 95% confidence intervals (CIs). A *p* value of < 0.05 was considered significant.

## Results

### Assessment of tumour extent (S-PCI) using preoperative CT scans (CT-PCI and CT-ascites) and CA-125

**Fig. 2:**
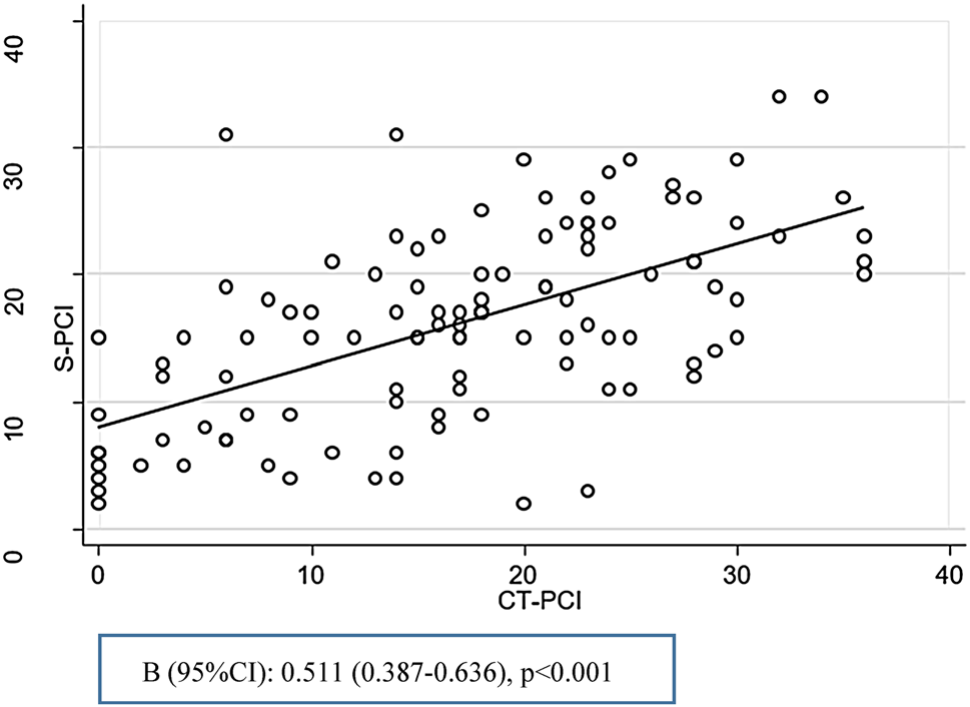
Linear regression analysis showing a positive correlation between increasing CT-PCI and S-PCI

Linear regression analysis revealed a positive correlation between increasing CT-PCI and the S-PCI (both continuous data) (0.511 (95% CI 0.387–0.639), p < 0.001) (Fig. 2). Statistical significance was maintained when the data were adjusted for CA-125 level, ascites and time between the CT scan and surgery (0.397 (95% CI 0.252–0.541) * p* < 0.001)) (Table 2).

**Table 2:** CT-PCI, CA-125, ascites, and the time interval between the CT examination and the surgery as predictive for tumour extent (S-PCI)

Variables	A		B	
	β (95% CI)	*p* value	β (95% CI)	*p* value
CT-PCI	0.511 (0.387–0.636)	< 0.001*	0.397 (0.252–0.541)	< 0.001*
log2 (CA-125)	1.439 (0.665–2.212)	< 0.001*	− 0.040 (− 0.840 to 0.760)	0.921
Days from CT to surgery		0.001*		0.021*
< 20	Ref		Ref	
20–39	6.019 (2.630–9.408)		3.163 (0.230–6.097)	
≥ 40	7.179 (3.277–11.080)		4.678 (1.260–8.097)	
Ascites CT (ml)		< 0.001*		0.038*
< 500	Ref		Ref	
500–1000	6.121 (0.807–11.436)		2.542 (− 2.147 to 7.231)	
≥ 1000	7.842 (4.288–11.395)		4.390 (1.027–7.753)	

*A* unadjusted analysis of each variable alone, *B* adjusted model including all variables in the table

*P < 0.05 was considered significant

When the ICC was used to compare CT-PCI and S-PCI divided into three groups (≤ 10, 11–20, ≥ 21), an agreement of 79% was found (0.580 (95% CI 0.442–0.0.991)).

No difference between the tumour extent of the different FIGO stages was found; the mean S-PCI was 16.31 for FIGO stage 3 and was 16.96 for FIGO stage IV (*p* = 0.711).

The amount of ascites was positively correlated with the S-PCI in both the crude and adjusted data (for ascites volume > 1000 ml: 4.390 (95% CI 1.027–7.753) *p* < 0.038) (Table 2). When S-ascites and CT-ascites were compared, the weighted kappa value was 0.678, indicating good conformity between the ascites volume estimated on CT scan and the intraoperative ascites volume (86% agreement).

The CA-125 level was related to tumour extent in the crude data (1.439 (0.665–2.212), *p* < 0.001), but when the data were adjusted for CT-PCI, ascites, and days between CT examination and surgery, no significant association between CA-125 and S-PCI was found (Table 2).

### The role of CT scans (CT-PCI, CT-ascites, CPLNs) and CA-125 in the preoperative evaluation of the risk of residual disease

The results from logistic regression models showed a significant association between CT-PCI and residual disease (OR 1.088 (1.037–1.142), *p* < 0.001).

**Fig. 3:**
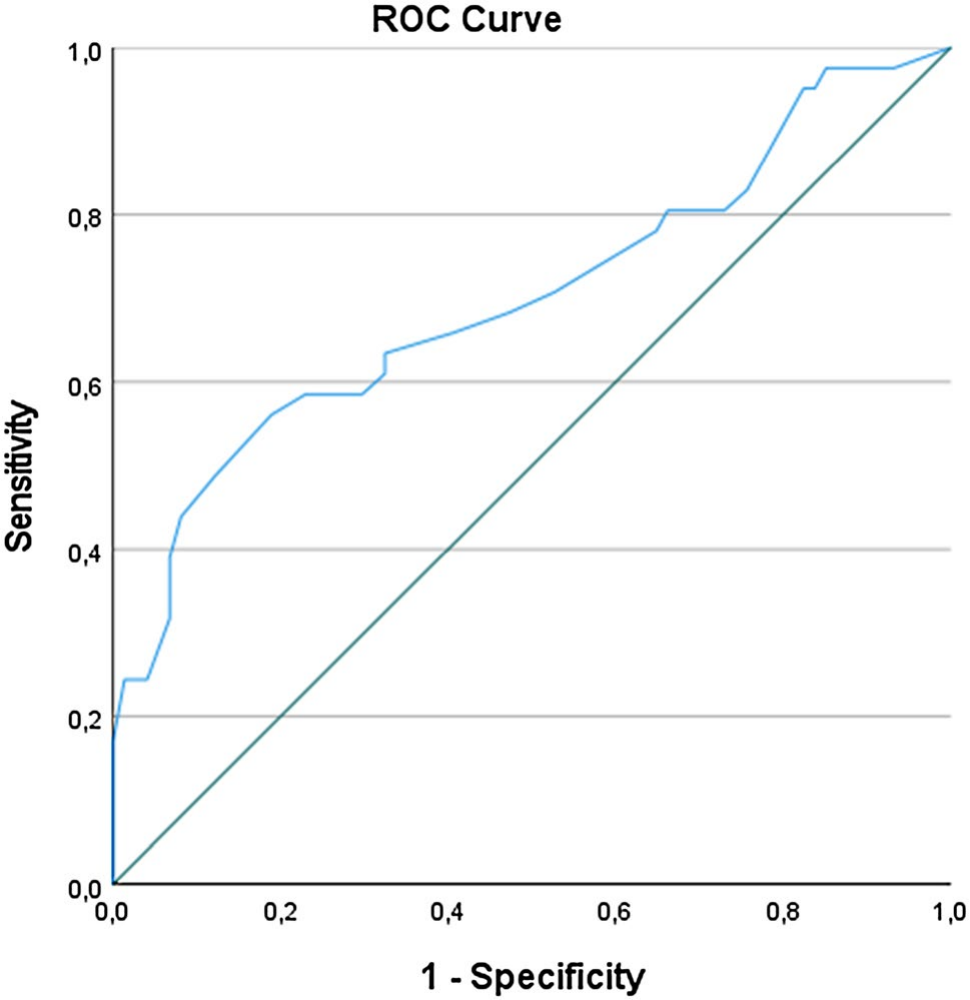
The ROC curve for the reported CT-PCI to residual disease

When the results were adjusted for CA-125 level, CPLNs, ascites, and days between the CT scan and surgery, the association was still significant (OR 1.069 (1.009–1.132), *p* < 0.023) (Table 3). The results were confirmed by ROC analyses, with an AUC of 0.715 (95% CI 0.609–0.822, *p* = 0.000) and generating a cut-off value of 21 for CT-PCI (Fig. 3). The sensitivity, specificity, positive predictive value and negative predictive value were 58.5% (CI 42.1–73.7%), 70.3% (CI 58.5–80.3%), 52.2% CI 41.3–62.7%) and 75.4% (CI 67.4–81.9%), respectively. The accuracy for CT-PCI ≥ 21 was 66.1% (56.67–74.65%).

**Table 3:** The relationship between CT-PCI and residual disease

	OR	95% CI	*p* value
CT-PCI	1.069	1.009–1.132	0.023*
log2(CA-125)	0.989	0.723–1.354	0.946
CT-CPLN	1.147	0.452–2.906	0.773
CT-ascites (ml) > 1000 ml	3.510	1.298–9.491	0.013*
*Days from CT to operation*			0.111
< 20	Ref.		
20–39	1.317	0.406–4.270	
≥ 40	3.456	0.950–12.578	

Adjusted for CA-125, CT-CPLN, ascites and days between CT scan and surgery

*P < 0.05 was considered significant

To identify a reason for unresectability, a group of 19 patients with residual disease ≥ 10 mm was analysed separately. The site of the residual disease and other indicated reasons for terminating the surgery were small intestinal carcinomatosis in 12 cases, and in 9 (75%) of these, intestinal carcinomatosis referred to abdominal regions from 9 to 12 (i.e., the small intestine) on the CT scan.

The preoperative volume of ascites, as measured by CT, was significantly associated with residual disease of any size (for ascites > 1000 ml (OR 3.510 (1.298–0-013), *p* < 0.013)) (Table 3).

A total of 50.4% (*n* = 60) of the patients exhibited CPLNs ≥ 5 mm in the short axis of the CT scan. In 46% (*n* = 56) of the patients, the dominant lymph node was located on the anterior side of the cardiophrenic space, whereas in 22% (*n* = 27) of the patients, it was located on the posterior side. Twenty-three patients had enlarged lymph nodes on both the anterior and posterior sides. A median of 1.17 (range 0–6) enlarged CPLNs was detected. The presence of CPLNs was not correlated with the amount of residual disease at the end of the surgery in any of the analyses (Table 3).

Every doubling of CA-125 increased the risk for residual disease by two times (OR 2.143 (0.97–4.605), although not significant in either unadjusted or adjusted results for CT-PCI, CPLN, ascites and days between diagnosis and surgery, *p* = 0.051 and *p* = 0.946, respectively (Table 3).

## Discussion

The preoperative estimation of tumour extent and its resectability are both of great importance to surgical outcome in AOC. In this study, both CT-PCI and CT ascites were correlated with the S-PCI, showing that CT might be a reliable tool in the preoperative assessment of tumour extent. The results are in accordance with previous studies on the estimation of tumour extent in ovarian cancer [20, 21]. In this study, when CT-PCI was high, the S-PCI was high; a one-unit increase in the CT-PCI corresponded to a 0.4-unit increase in the S-PCI. In the non-CCS group, the number of stage IV patients did not significantly differ from that of patients without residual disease, indicating that the surgical effort seemed to be at the same level when treating patients in both stages.

The S-PCI was related to the time interval between the CT scan and surgery. Patients with a longer time interval than the reference of 20 days had higher S-PCI scores. This can be explained by tumour progression or a higher tumour load from the beginning, leading to impaired nutritional status that requires a longer preoperative rehabilitation time and a prolonged time between the CT scan and surgery. A future recommendation for the time lapse between CT and surgery might be warranted.

A good correlation was also found between the preoperative estimation of ascites volume and S-PCI, indicating that patients with CT ascites above 1000 ml had a 4.4-times higher risk of an increased S-PCI score, indicating a more pronounced tumour extent.

Residual disease after primary debulking surgery is one of the most important prognostic factors for survival in AOC [22]. This study intended to elucidate whether patients with residual disease could be identified preoperatively. CT-PCI correlated well with the risk of residual disease, which is in agreement with other studies. Avesani et al. [23] found a strong correlation between the CT-PCI and residual disease of any size at the end of the surgery.

Llueca et al. [24] tried to establish a predictive model for unresectability using CT, laparoscopy, and laparotomy. Their best cut-off for predicting suboptimal cytoreductive surgery was PCI > 20 for the three diagnostic techniques, with 91% specificity and 27% sensitivity for the CT scan [24]. Jönsdottir et al. [25] studied the correlation between the S-PCI and surgical outcome and found a PCI cut-off of 24. In their study, 62% of patients with PCI above 24 had an unsatisfactory surgical outcome defined as suboptimal cytoreductive surgery (residual disease ≥ 10 mm). The authors concluded that neoadjuvant chemotherapy could be considered if the PCI is higher than 24 [25]. Since S-PCI is an intraoperative observation, the patients need to have anaesthesia and at least laparoscopy to record the PCI score. This implies a two-step method or open/closed surgery, and both procedures are inferior to preoperative accurate scoring. In our study, we focussed on the preoperative evaluation to minimize the number of unnecessary laparotomies.

CT may both overestimate and underestimate the true peritoneal carcinomatosis amount [26]. Technical challenges, amount of intraperitoneal fat, size of peritoneal lesions, fibrosis and ascites may all challenge a true estimation of the peritoneal carcinomatosis [27, 28]. When PCI was divided in three groups, a moderate agreement between the groups was founded. In this study, certain underestimation may be present when the tumour burden is low and an overestimation when the tumour burden is high, as showed in Table 1. The clinical implication of underestimation of CT-PCI might result in a higher number of open/close procedures. However, this happens at the lower PCI scores of 11–20 where surgery usually is the first choice. An overestimation can risk refrain of surgery with increased neoadjuvant therapy rates.

Some studies have analysed separate regions and found that PCI in certain areas is more related to unresectability. Abdominal regions 9–12, corresponding to the small intestine, were significantly more predictive of residual disease than the entire PCI [29]. The same trend was found in the present study. In the patient group with residual disease above 10 mm, 12 patients exhibited small intestinal carcinomatosis. Nine (75%) of them had small intestinal carcinomatosis, correctly predicted by the CT scan. We consider that centralization of cancer care can improve not only surgical competence but also radiological competence. If the same specialist radiology team had interpreted the CT scan, all nine patients (75% patients with residual disease above 10 mm) might have avoided aggressive surgery with an uncertain survival benefit [30]. On the other hand, there is no clear-cut evidence for whether neoadjuvant chemotherapy with complete resection or upfront surgery with residual tumour < 5 mm is the most suitable method of treatment. The ongoing Trial of Radical Upfront Surgical Therapy (TRUST) study might provide an answer in a few years [31].

The quantification of ascites has, to our knowledge, been sparsely evaluated in preoperative CT scans in patients with AOC. In this study, the CT-based evaluation of ascites volume correlated with the ascites volume determined intraoperatively. When ascites is quantified intraoperatively, large volumes correlate with unresectability and worse PFS and OS [32, 33]. Massive ascites evaluated by CT scan correlated with an increased risk of residual tumour in the present study. A three- and half-fold increased risk for residual disease in ascites quantified with CT to be over 1000 ml was found.

CPLNs in AOC are associated with carcinomatosis in the upper abdomen, diaphragmatic carcinomatosis, and a worse prognosis regardless of surgical removal during cytoreductive surgery. Of the 350 patients analysed by Prader et al. [13], almost 40% had negative CPLNs, while the rest had radiologically positive CPLNs. In patients with macroscopically complete tumour resections, CPLN metastases were associated with reduced PFS and OS. Nevertheless, the role of CPLN resection remains unclear [13]. In this study, no correlation was found between the presence of CPLNs on CT images and residual disease.

The relationship between CA-125 level and surgical outcome is controversial. Many studies have found a correlation between CA-125 > 500 U/mL and suboptimal cytoreduction [15, 16, 18, 34, 35]. The data analysed in the present study indicated a close to significance relationship between CA-125 level and the amount of residual disease in the unadjusted data. When the data were adjusted for CT-PCI, ascites, and CPLN, no significant association between CA-125 level and residual disease was found.

The strength of this study is that it was conducted in a single tertiary centre with a high CCS rate and highly specialized radiology department, both of which increase the accuracy of CT-PCI and S-PCI scoring. The fact that the patients’ clinical records, including their histopathological records, are all connected to one entity, thus making it possible to verify PCI as true carcinosis, can be considered a strength as well.

Interreader variability was not addressed which is a limitation of the study. However, the image evaluation may be seen as a consensus evaluation made by the referring hospital radiologist and the multidisciplinary conference radiologist. Another limitation of this study is the retrospective nature of PCI documentation and the relatively small cohort of patients.

## Conclusions

Numerical estimation of the tumour extent by CT is feasible in AOC but may require a skilled radiologist. Together with a high volume of ascites, as shown in this study, the CT-PCI might be used as an indicator for residual disease in upfront surgery for AOC. Further prospective studies are needed.

## Data Availability

The datasets used and/or analysed in the present study are available from the corresponding author upon reasonable request.
